# Effects of a Computerized Training on Attentional Capacity of Young Soccer Players

**DOI:** 10.3389/fpsyg.2019.02279

**Published:** 2019-10-21

**Authors:** Rafael E. Reigal, Fernando González-Guirval, Juan P. Morillo-Baro, Verónica Morales-Sánchez, Rocío Juárez-Ruiz de Mier, Antonio Hernández-Mendo

**Affiliations:** ^1^University of Malaga, Malaga, Spain; ^2^Department of Social Psychology, Social Work, Anthropology and East Asian Studies, University of Málaga, Málaga, Spain; ^3^Department of Evolutionary Psychology and Education, University of Málaga, Málaga, Spain

**Keywords:** attention, cognitive functioning, soccer, computerized training, sport

## Abstract

The purpose of this work was to analyze the effects of a computerized training on attentional capacity in a group of young soccer players. Seventy-five male adolescents from two soccer clubs in the city of Malaga (Spain) and aged between 14 and 18 (15.45 ± 1.43 years) participated in the investigation. A quasi-experimental design was used, and the adolescents were divided into control (*n* = 38) and experimental (*n* = 37) groups. The experimental group underwent a computerized training (Rejilla 1.0) of their attention during 9 weeks and 27 sessions. In addition, the D2 attention test was used to analyze the evolution of participants after the intervention program. The results showed positive effects of the computerized intervention program on selective attention, observing changes both in the executions of the software used (*p* < 0.001, Cohen's *d* = 1.58, 95% CI [1.06, 2.11]) and in the main measures of the D2 test, total effectiveness (*p* < 0.001, Cohen's *d* = 0.62, 95% CI [0.15, 1.08]) and concentration (*p* < 0.01, Cohen's *d* = 0.48, 95% CI [0.02, 0.94]).

## Introduction

Scientific literature has highlighted in recent years that cognitive functioning of athletes could be a determinant of their performance and predictor of their level of expertise (Romeas et al., 2016; Fink et al., 2018). Some research has indicated that athletes who show better cognitive functioning show increases in performance, especially in those disciplines that require continuous adaptation to play and a great ability to anticipate and maintain attention (Verburgh et al., 2014). For example, Huijgen et al. (2015) observed in a group of elite soccer players between 13 and 17 years old better scores in inhibitory control and cognitive flexibility than in a group of sub-elite soccer players. Verburgh et al. (2014) compared two groups of soccer players between 8 and 16 years old, indicating better scores on inhibitory control and the ability to reach and maintain alertness in the most talented group. Vestberg et al. (2017) investigated a group of soccer players between 12 and 19 years old, revealing that executive functions are cognitive abilities that predict sport success.

Specifically, some works have highlighted that aspects such as attention and concentration are significantly related to the behavior of athletes (Weinberg and Gould, 2010; Carraça et al., 2018; Love et al., 2018). Among existing research, Williams et al. (2011) revealed that visual attention training may influence performance in sports modalities in which moving objects such as a ball are used. Roca et al. (2018) analyzed 44 soccer players with an average age of 20.8 years old and observed that creativity shown in the decision making before different simulated game actions was modulated by attentional aspects. Thus, they indicated that a greater capacity to attend to the stimuli presented was a determinant to present more creative solutions during the game.

Attention has been widely explored in numerous works and is considered a fundamental cognitive capacity for humans, because it allows to select the necessary information and to facilitate a correct functioning (Desimone and Duncan, 1995; Chun et al., 2011; Rosenberg et al., 2017). In addition, the development of attentional capacity is linked to other dimensions of cognitive functioning, such as memory, executive control, or learning (Logue and Gould, 2014; Bialystok, 2015; Campillo et al., 2018). For all these reasons, the evaluation and development of attention have been the object of interest in different social areas such as relevant as clinical, educational, work, or sports area (Memmert et al., 2009; Memmert, 2011; Gray et al., 2016; Kirk et al., 2016; Spaniol et al., 2018).

Attention is a complex construct that has different manifestations such as attentional, selective, serial, divided, or sustained span, among others (Posner and Petersen, 1990; Estévez-González et al., 1997). Specifically, one of the dimensions that have aroused the most interest among researchers is selective attention, which would refer to the ability to attend to specific stimuli and ignore others, which is very relevant to adapt to multiple tasks and functioning adequately in contexts such as education or sports (Estévez-González et al., 1997; Bar-Eli et al., 2011; Giuliano et al., 2014). For example, it is considered that in soccer, this capacity is involved in habitual processes during the game, such as determining to which player the ball has to be passed to or which movements of both teammates and opponents are decisive for the development of a game (Romeas et al., 2016; Gonçalves et al., 2017).

Research has shown that attention can be trained in populations of different characteristics and through different methods (Wass et al., 2011; Posner et al., 2015; Olfers and Band, 2018). Among others, positive effects of systematic training of this cognitive capacity in people with generalized social phobia, anxiety, autism, traumatic brain injury, or attention deficit hyperactivity have been indicated (Amir et al., 2009; Christiansen and Oades, 2010; Bar-Haim et al., 2011; Powell et al., 2016; Séguin et al., 2018). Likewise, different strategies have been used in sport to train perceptive and attentional processes with the aim of improving the response of athletes to different game situations and of trying to increase their performance (Calmels et al., 2004; Romeas et al., 2016).

In recent years, the use of computerized tools for attention assessment and training has increased (Hernández-Mendo and Ramos-Pollán, 1995a,b; Reid et al., 2009; Rabiner et al., 2010; Chamberlain et al., 2011; Hernández-Mendo et al., 2012; Montani et al., 2014; Bogdanova et al., 2016; Kirk et al., 2016). The development of technology and adaptation of instruments to digital environments has increased the resources to analyze cognitive functioning in people. This type of tool offers greater versatility for collecting and handling information, processing stored data, adapting its operation to the performer, or modifying characteristics of the exercise that may increase motivation during the performance of the exercises (González de la Torre and González de la Torre, 2003; Fernández-Calvo et al., 2011).

Based on the precedents described, the purpose of this work is to analyze the effects of a computerized training program of selective attention through the software Rejilla 1.0. 4 (Hernández-Mendo et al., 2012) in a group of young soccer players (see Supplementary Video S1).

## Methods

### Sample

Seventy five male teenagers and young men from two soccer clubs in Malaga (Spain), aged between 14 and 18 years old (*M* ± SD: age = 15.45 ± 1.43 years old), took part in this research. Participants trained 3 or 4 days/week, lasting ~90 min per session. Everyone had at least 5 years of soccer practice experience. The exclusion criteria were physical and psychological health problems that could affect the research or no informed consent (no participant had to be suppressed). The sample was organized into two groups: control (they did not participate in the attention training program and did not perform alternative tasks) and experimental (they participated in the intervention program at times other than sports training).

### Instruments and Measures

#### Rejilla v. 1.0

**Figure 1:**
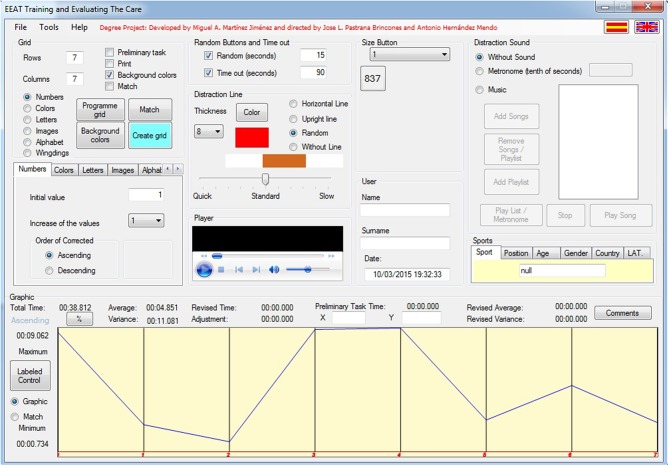
Main screen of the program Rejilla 1.0.

This program is described in Hernández-Mendo et al. (2012). The program is a Windows desktop application made under the .NET platform in the C# programming language and with the Visual Studio programming environment. The program is downloaded from the online evaluation platform MenPas (www.menpas.com) (González-Ruiz et al., 2010, 2018). When the application is started, an initial screen appears requesting the user name and password for the MenPas platform. The main screen of the program appears in Figure 1 (see Supplementary Material). This program can work with six types of stimuli (numbers, colors, letters, images, alphabet, and windings) and, depending on the programming of the time and that is performed with/without pairing, can work with different types of attention. It is possible to program the sizes of stimuli and background colors and to change place by time intervals, presentation times, and the use of distracting stimulus such as lines (you can program the color and width), sounds, or metronome. It allows to know the time between each stimulus and the latency times. It calculates efficiency and effectiveness indicators as well as percentages of relative/absolute hits/errors. The main type of attention that can be evaluated and trained with this software is selective or focal attention/serial attention (Estévez-González et al., 1997). You can also train/sustained assessment when no time limits are set or it is set at 15 min or more. One could also work on attention to visual hemifield displacement (Estévez-González et al., 1997) using the pairing option. For this study, the hits and errors of each task have been recorded.

#### D2 Attention Test (Brickenkamp, 2002)

It is a test that is used to explore the capacity to attend to the relevant stimulus of a task in a fast and precise way, ignoring the irrelevant ones, being considered a manifestation of selective attention and concentration. The test is based on discriminating between 47 characters in each of the 14 rows, with a total of 658 elements. You have 20 s to make each row. Stimulus contains the letters “d” or “p,” which may be accompanied by one or two stripes at the top of the item, at the bottom, or both. In order to perform the test properly, the “d” must be crossed out with two lines (regardless of position), considered as relevant stimulus. The test is always carried out from left to right and from top to bottom. The scores that can be obtained are as follows: TA (total number of attempts), TH (total number of hits), O (omissions or number of relevant stimuli not crossed out), C (omissions or errors), TET (total effectiveness in the test = TA—[O + C]), CON (concentration = TH—C), TA+ (last stimulus analyzed in the row with the most attempted elements), TA– (last stimulus analyzed in the row with the fewest attempted elements), and VAR (index of variation between the last stimulus analyzed between different rows = [TA+]—[TA–]). This test possesses a test–retest reliability in the original study superior to 0.90.

### Procedure

The sports clubs were contacted, and permission from the sports management and coaches was obtained to carry out the research. In addition, informed and written consent was achieved to participate in the study (for those athletes under-18, consent from parents or legal guardians was required). In addition, authorization was also obtained from the Ethics Committee of the University of Malaga (CEUMA, no. 243, 19-2015-H), and the ethical principles of the Declaration of Helsinki (World Medical Association, 2013) were respected during the research process.

Two evaluations were carried out for D2 attention test, initial and final, and three assessments for Rejilla 1.0 software, initial, midterm, and final. The computerized exercises (Rejilla 1.0) consisted of eight tasks: (1) cancellation of numbers (in pairs and without disappearing) in color matrix, 10 × 10 size, and distracting line; (2) cancellation of numbers (in pairs and disappearing) in color matrix, 10 × 10 size, and distracting line; (3) cancellation of numbers (no pairs and without disappearing) in color matrix, 10 × 10 size, and distracting line; (4) cancellation of numbers (no pairs and disappearing) in color matrix, 10 × 10 size, and distracting line; (5) cancellation of numbers (in pairs and without disappearing) in color matrix, 11 × 11 size, and distracting line; (6) cancellation of numbers (in pairs and disappearing) in color matrix, 11 × 11 size, and distracting line; (7) cancellation of numbers (no pairs and without disappearing) in color matrix, 11 × 11 size, and distracting line; and (8) cancellation of numbers (no pairs and disappearing) in color matrix, 11 × 11 size, and distracting line.

**Figure 2:**
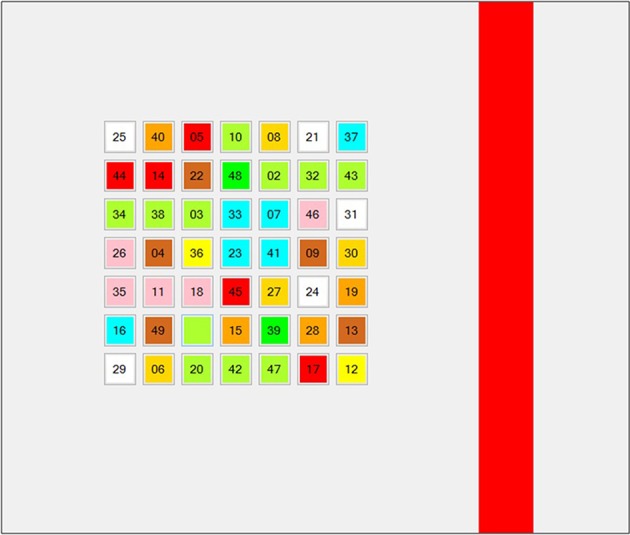
Example of task (cancellation of numbers (in pairs and disappearing) in color matrix, 7 × 7 size, and distracting line).

During the intervention, the experimental group was involved in attention training programs using Rejilla 1.0 software, 3 days/week during 9 weeks. In each session, they performed eight tasks: (1) cancellation of numbers (in pairs and disappearing) in color matrix, 7 × 7 size, and distracting line (Figure 2); (2) cancellation of numbers (no pairs and without disappearing) in color matrix, 7 × 7 size, and distracting line; (3) cancellation of numbers (in pairs and disappearing) in color matrix, 8 × 8 size, and distracting line; (4) cancellation of numbers (no pairs and without disappearing) in color matrix, 8 × 8 size, and distracting line; (5) cancellation of numbers (in pairs and disappearing) in color matrix, 9 × 9 size, and distracting line; (6) cancellation of numbers (no pairs and without disappearing) in color matrix, 9 × 9 size, and distracting line; (7) cancellation of numbers (in pairs and disappearing) in color matrix, 10 × 10 size, and distracting line; and (8) cancellation of numbers (no pairs and without disappearing) in color matrix, 10 × 10 size, and distracting line. The control group did not participate in the attention training program and did not perform alternative tasks, although they did continue training soccer.

### Data Analysis

Descriptive and inferential analyses were used to process the information collected. The values of mean, standard deviation, skewness, kurtosis, and *Shapiro–Wilk* test were obtained. Intragroup means were compared using the Friedman and Wilcoxon tests. Intergroup means were compared through the Mann–Whitney *U*-test. Cohen's *d* statistic was performed to calculate the effect size. Data were analyzed with SPSS statistical program (SPSS Inc. v.24.0, Chicago, IL, USA).

## Results

Tables 1, 2 show descriptive and normal statistics for initial, intermediate, and final assessments in Rejilla 1.0 tests and for both groups. As can be seen, the results indicate that the distributions did not meet the criterion of normality in most cases.

**Table 1:** Hits and errors (mean and standard deviation) in Rejilla 1.0 tests for both groups and differences between groups.

	**Control group**			**Experimental group**		
	**Initial**	**Midterm**	**Final**	**Initial**	**Midterm**	**Final**
	***M* ± *SD***	***M* ± *SD***	***M* ± *SD***	***M* ± *SD***	***M* ± *SD***	***M* ± *SD***
H1	1.97 ± 1.15	2.05 ± 1.35	2.26 ± 1.37	2.27 ± 1.54	2.97 ± 1.72^a^	4.16 ± 2.05^c^
H2	2.84 ± 1.44	2.08 ± 1.26	2.29 ± 1.74	2.62 ± 1.21	2.70 ± 1.60	4.03 ± 1.85^c^
H3	2.21 ± 1.23	2.55 ± 1.57	2.16 ± 1.24	2.89 ± 1.71	3.65 ± 2.20^a^	5.92 ± 2.65^c^
H4	2.79 ± 1.83	2.87 ± 1.61	2.58 ± 1.08	4.00 ± 2.08	3.73 ± 2.38	6.38 ± 3.10^c^
H5	2.16 ± 1.44	1.87 ± 0.93	1.76 ± 0.91	2.05 ± 1.31	3.57 ± 2.43^c^	4.11 ± 1.79^c^
H6	2.37 ± 1.20	1.79 ± 0.78	1.79 ± 1.09	2.35 ± 1.46	2.62 ± 1.77	4.19 ± 1.90^c^
H7	2.05 ± 1.14	1.87 ± 1.02	2.47 ± 1.69	2.81 ± 1.76	2.73 ± 1.94^a^	5.24 ± 2.62^c^
H8	2.55 ± 1.39	2.29 ± 1.21	2.37 ± 1.89	2.89 ± 1.54	3.14 ± 2.20	5.97 ± 2.93^c^
T (H)	2.37 ± 0.64	2.17 ± 0.61	2.21 ± 0.84	2.74 ± 0.91	3.14 ± 1.44^c^	5.01 ± 1.81^c^
E1	2.37 ± 3.19	2.55 ± 3.27	2.61 ± 3.45	0.92 ± 1.09^a^	1.11 ± 1.24	1.27 ±0.99
E2	2.00 ± 2.94	1.82 ± 2.64	2.47 ± 3.34	1.22 ± 1.51	1.08 ± 1.09	0.97 ±0.96
E3	2.87 ± 3.50	2.37 ± 3.14	2.39 ± 3.08	2.22 ± 2.63	1.62 ± 2.34	0.70 ± 1.51^b^
E4	1.29 ± 2.73	2.32 ± 3.35	1.58 ± 2.56	0.89 ± 1.98^b^	0.81 ± 1.93^a^	0.38 ±0.64^a^
E5	2.11 ± 3.06	1.76 ± 2.75	2.24 ± 2.97	1.32 ± 1.97	0.81 ± 1.10	1.19 ± 1.08
E6	1.37 ± 2.33	1.95 ± 2.75	2.63 ± 3.55	1.11 ± 1.76	0.76 ± 0.98	0.95 ± 1.22
E7	2.26 ± 3.37	2.61 ± 3.37	2.34 ± 3.16	1.46 ± 2.73	1.38 ± 2.19	0.59 ± 1.28^c^
E8	1.61 ± 2.80	1.61 ± 2.88	1.05 ± 2.60	0.76 ± 1.55	0.59 ± 1.72	0.59 ± 1.01^a^
T (E)	1.98 ± 2.35	2.12 ± 2.16	2.16 ± 2.29	1.24 ± 1.22	1.02 ± 0.96	0.83 ± 0.46^a^

*M, mean; SD, standard deviation; H, hits; E, errors; T, total*.

Cross-group:

a p < 0.05;

b p < 0.01;

c *p < 0.001*.

**Table 2:** Skewness, kurtosis, and Shapiro–Wilk tests for both groups and different Rejilla 1.0 tasks.

	**Initial**			**Midterm**			**Final**		
	***S***	***K***	***S-W***	***S***	***K***	***S-W***	***S***	***K***	***S-W***
**CONTROL GROUP**									
H1	1.51	2.79	0.79^***^	2.59	9.50	0.70^***^	1.56	2.85	0.81^***^
H2	0.29	−0.87	0.91^**^	1.64	3.20	0.77^***^	3.63	17.14	0.61^***^
H3	1.67	4.79	0.81^***^	1.07	0.90	0.85^***^	1.38	1.91	0.81^***^
H4	0.88	0.16	0.87^***^	1.45	2.40	0.84^***^	1.61	3.79	0.78^***^
H5	2.04	4.23	0.71^***^	1.11	1.67	0.80^***^	0.95	−0.04	0.78^***^
H6	0.83	0.68	0.88^***^	0.76	0.28	0.81^***^	2.01	5.07	0.72^***^
H7	0.94	−0.05	0.82^***^	1.41	1.79	0.77^***^	2.00	5.34	0.78^***^
H8	0.49	−0.98	0.87^***^	0.67	−0.47	0.86^***^	3.01	11.43	0.65^***^
T (A)	0.12	−0.88	0.98	-0.23	−1.11	0.95	2.51	9.63	0.77^***^
E1	1.72	1.66	0.68^***^	1.36	0.60	0.75^***^	1.28	0.24	0.74^***^
E2	1.83	2.49	0.69^***^	1.72	2.21	0.72^***^	1.49	1.03	0.73^***^
E3	1.25	0.25	0.76^***^	1.34	0.66	0.74^***^	1.42	0.98	0.76^***^
E4	2.74	6.69	0.51^***^	1.41	0.59	0.71^***^	1.98	3.37	0.68^***^
E5	1.97	2.70	0.65^***^	1.63	1.74	0.70^***^	1.39	0.71	0.75^***^
E6	2.92	8.98	0.59^***^	1.81	2.64	0.72^***^	1.36	0.33	0.71^***^
E7	1.47	0.79	0.69^***^	1.41	0.70	0.74^***^	1.74	1.85	0.69^***^
E8	2.24	4.52	0.62^***^	2.01	3.03	0.61^***^	2.86	7.37	0.46^***^
T (E)	2.47	6.00	0.65^***^	1.42	1.49	0.81^***^	1.60	1.82	0.78^***^
**EXPERIMENTAL GROUP**									
H1	1.26	1.09	0.80^***^	0.73	0.30	0.90^***^	0.63	0.19	0.94^*^
H2	0.30	−1.02	0.89^**^	0.56	−0.93	0.87^***^	0.43	−0.35	0.93^*^
H3	0.95	0.58	0.88^***^	1.10	0.61	0.87^***^	-0.17	−0.68	0.96
H4	1.25	2.33	0.90^**^	1.72	3.15	0.80^***^	0.27	−0.68	0.95
H5	1.07	0.01	0.78^***^	2.34	8.45	0.79^***^	0.66	0.45	0.94
H6	1.11	0.52	0.83^***^	1.28	1.44	0.82^***^	0.51	−0.17	0.95
H7	0.95	0.11	0.87^***^	2.53	8.70	0.74^***^	-0.07	−1.00	0.95
H8	0.33	−0.95	0.91^**^	1.26	1.49	0.86^***^	-0.10	−0.65	0.94
T (H)	0.83	1.70	0.94^*^	1.94	4.92	0.83^***^	0.10	−1.49	0.91^**^
E1	1.67	4.11	0.77^***^	1.90	5.47	0.76^***^	-0.04	−1.23	0.84^***^
E2	1.86	3.92	0.74^***^	0.78	−0.05	0.85^***^	0.86	0.94	0.82^***^
E3	1.50	1.36	0.78^***^	2.13	4.68	0.71^***^	3.63	15.63	0.52^***^
E4	2.87	8.00	0.51^***^	3.48	14.34	0.48^***^	1.50	1.15	0.62^***^
E5	2.73	9.77	0.68^***^	1.58	2.22	0.74^***^	0.59	−0.25	0.87^***^
E6	3.25	12.11	0.57^***^	2.38	8.64	0.69^***^	1.45	2.01	0.77^***^
E7	2.11	3.49	0.60^***^	2.22	4.42	0.64^***^	3.86	17.86	0.50^***^
E8	3.30	13.12	0.55^***^	4.84	26.00	0.38^***^	1.76	2.68	0.65^***^
T (E)	2.33	7.03	0.75^***^	2.46	6.98	0.72^***^	1.21	1.12	0.89^**^

*S, skewness; K, kurtosis; S-W, Shapiro–Wilk; H, hits; E, errors; T, total*.

* p < 0.05;

** p < 0.01;

*** *p < 0.001*.

**Figure 3:**
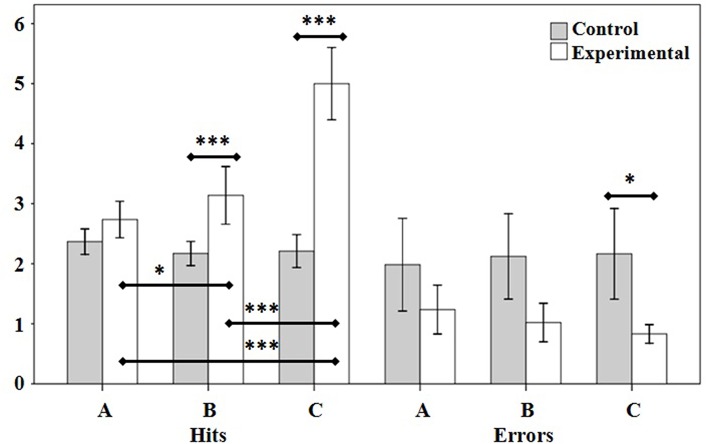
Intragroup and intergroup scores and comparisons for the initial, intermediate, and final measures of hits and errors (*M* ± DT) of the Rejilla 1.0 exercise scores. A, initial assessment; B, midterm assessment; C, final assessment. *p < 0.05; ***p < 0.001.

Table 3 shows the comparisons between the different assessments in each group. As it can be observed, the scores in hits for the experimental group manifested greater differences between evaluations than for the control group. For total scores, there were differences in the experimental group in initial vs. midterm (*p* < 0.05), midterm vs. final (*p* < 0.001), and initial vs. final assessment (*p* < 0.001). Figure 3 shows the average hit and error scores of the Rejilla 1.0 exercises used.

**Table 3:** Differences between evaluations (Friedman and Wilcoxon) in both groups for Rejilla 1.0 tests (control and experimental).

	**Control group**				**Experimental group**			
	**Friedman (χ^**2**^)**	**Assessments**			**Friedman (χ^**2**^)**	**Assessments**		
		**A vs. B**	**B vs. C**	**A vs. C**		**A vs. B**	**B vs. C**	**A vs. C**
H1	0.82	–	–	–	21.76^***^	<0.05	<0.05	<0.001
H2	4.79	–	–	–	21.31^***^	–	<0.001	<0.001
H3	0.67	–	–	–	21.11^***^	–	<0.001	<0.001
H4	0.76	–	–	–	18.31^***^	–	<0.001	<0.01
H5	1.56	–	–	–	27.24^***^	<0.01	–	<0.001
H6	7.11^*^	<0.05	–	<0.05	19.89^***^	–	<0.001	<0.001
H7	7.13^*^	–	<0.05	–	20.44^***^	–	<0.001	<0.001
H8	0.15	–	–	–	28.01^***^	–	<0.001	<0.001
T (H)	0.51	–	–	–	36.02^***^	<0.05	<0.001	<0.001
E1	0.44	–	–	–	2.09	–	–	–
E2	3.04	–	–	–	1.09	–	–	–
E3	1.53	–	–	–	9.65^**^	–	–	<0.01
E4	0.54	–	–	–	0.07	–	–	–
E5	1.58	–	–	–	2.34	–	–	–
E6	2.71	–	–	–	0.38	–	–	–
E7	1.87	–	–	–	3.55	–	–	–
E8	3.10	–	–	–	0.58	–	–	–
T (E)	0.18	–	–	–	1.70	–	–	–

*A, initial assessment; B, midterm assessment; C, final assessment*.

* p < 0.05;

** p < 0.01;

*** *p < 0.001*.

Table 4 shows the descriptive and normality statistics (Shapiro–Wilk) for the pre-measurements and post-measurements of D2 attention test for both groups. As can be seen, the results indicate that the distributions did not meet the criterion of normality in many scores.

**Table 4:** Descriptions and normality test (*Shapiro–Wilk*) of the values obtained in the D2 test measurements.

	**Pre-test**					**Post-test**				
	***M***	***SD***	***S***	***K***	***S-W***	***M***	***SD***	***S***	***K***	***S-W***
**CONTROL GROUP**										
D2-TA	59.50	17.28	0.19	−1.08	0.95	65.68	20.29	−0.31	−0.67	0.93^*^
D2-TH	58.16	20.37	−0.03	−0.42	0.94	62.63	21.92	0.16	−0.98	0.93^*^
D2-O	43.26	19.87	−0.39	−0.34	0.96	46.18	22.41	−0.11	−1.27	0.92^*^
D2-C	43.50	20.25	0.33	−0.33	0.93^*^	45.21	27.78	0.77	0.26	0.90^**^
D2-TET	65.42	20.66	−0.03	−0.77	0.95	68.47	20.94	−0.12	−0.90	0.91^**^
D2-CON	60.79	25.25	−0.10	−1.23	0.90^**^	64.11	23.60	−1.11	0.23	0.79^***^
D2-(TA+)	61.03	17.08	−0.51	−1.15	0.88^**^	66.58	16.60	−0.97	−0.01	0.84^**^
D2-(TA–)	63.84	20.63	−0.52	−0.96	0.89^**^	67.74	22.85	−1.00	0.01	0.83^**^
D2-VAR	54.66	21.83	−0.01	−0.54	0.98	56.47	22.44	0.06	−0.71	0.96
**EXPERIMENTAL GROUP**										
D2-TA	63.65	19.98	−0.01	−1.34	0.91^**^	72.51	25.33	−1.73	2.87	0.73^***^
D2-TH	65.73	22.00	−0.36	−0.47	0.93^*^	75.86	23.77	−1.72	2.74	0.75^***^
D2-O	45.84	21.46	0.14	−0.19	0.98	47.95	21.13	0.06	−0.92	0.95
D2-C	42.59	28.32	0.86	−0.52	0.84^***^	44.97	29.06	1.25	1.21	0.84^***^
D2-TET	66.95	20.79	−0.09	−1.15	0.91^**^	78.84	17.65	−1.44	4.82	0.82^***^
D2-CON	63.05	22.64	−1.28	0.08	0.68^***^	73.65	21.30	−3.40	12.13	0.42^***^
D2-(TA+)	63.27	17.74	−1.50	1.76	0.74^***^	67.38	16.64	−2.98	9.50	0.53^***^
D2-(TA–)	68.57	21.61	−1.35	0.85	0.77^***^	74.49	21.73	−1.87	2.37	0.60^***^
D2-VAR	48.22	27.01	0.33	−0.99	0.92^*^	49.76	29.13	0.94	0.24	0.88^**^

*S, skewness; K, kurtosis; D2, D2 test; TA, total number of attempts; TH, total number of hits; O, omissions; C, commissions; TET, total effectiveness in the test; CON, concentration index; (TA+), last stimulus analyzed in the row with the most attempted elements; (TA–), last stimulus analyzed in the row with the fewest attempted elements; VAR, variation index*.

* p < 0.05;

** p < 0.01;

*** *p < 0.001*.

**Figure 4:**
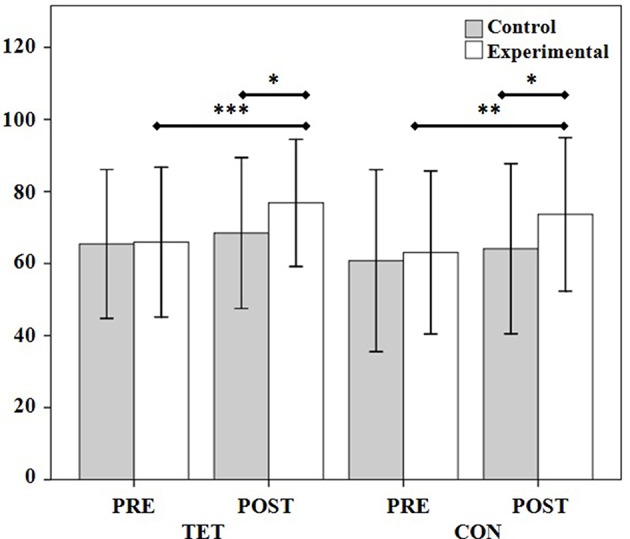
Intragroup and intergroup scores and comparisons for the pretest and posttest measures of the main measures of the D2 test (TET and CON). TET, total effectiveness in the test; CON, concentration index. *p < 0.05; **p < 0.01; ***p < 0.001.

Table 5 shows intergroup and intragroup differences between D2 attention test measures. As can be seen, the differences between the groups increased after the training program in TH (*p* < 0.05, Cohen's *d* = 0.58, 95% CI [0.12, 1.04]), TET (*p* < 0.05, Cohen's *d* = 0.43, 95% CI [−0.03, 0.89]) and CON (*p* < 0.05, Cohen's *d* = 0.42, 95% CI [−0.03, 0.88]) (Figure 4). Significative increases were observed in both groups in TH (control: *p* < 0.05, Cohen's *d* = 0.21, 95% CI [−0.24, 0.66]; experimental: *p* < 0.05, Cohen's *d* = 0.44, 95% CI [−0.02, 0.91]) but not in the experimental group in the measures TA (*p* < 0.01, Cohen's *d* = 0.39, 95% CI [−0.07, 0.85]), TET (*p* < 0.001, Cohen's *d* = 0.62, 95% CI [0.15, 1.08]), CON (*p* < 0.01, Cohen's *d* = 0.48, 95% CI [0.02, 0.94]) (Figure 4), TA+ (*p* < 0.05, Cohen's *d* = 0.24, 95% CI [−0.22, 0.69]), and TA– (*p* < 0.05, Cohen's *d* = 0.27, 95% CI [−0.18, 0.73]).

**Table 5:** Comparisons between intergroups and intragroups.

	***Group***		***Factor***	
	***Control* Pre vs. post**	***Experimental* Pre vs. post**	***Pretest* C vs. E**	***Posttest* C vs. E**
D2-TA	−1.87	−2.87^**^	–.77	−1.55
D2-TH	−2.07^*^	−2.44^*^	−1.73	−2.56^*^
D2-O	−0.22	−0.83	−0.30	−0.76
D2-C	−0.12	−0.14	−0.59	−0.93
D2-TET	−1.10	−3.65^***^	−0.26	−2.08^*^
D2-CON	−0.98	−3.11^**^	−0.16	−2.01^*^
D2-TA+	−1.74	−1.97^*^	−0.55	−0.51
D2-TA–	−1.59	−2.21^*^	−1.55	−1.60
D2-VAR	−0.25	−0.50	−1.15	−1.24

*D2, D2 test; TA, total number of attempts; TH, total number of hits; O, omissions; C, commissions; TET, total effectiveness in the test; CON, concentration index; (TA+), last stimulus analyzed in the row with the most attempted elements; (TA–), last stimulus analyzed in the row with the fewest attempted elements; VAR, variation index*.

* p < 0.05;

** p < 0.01;

*** *p < 0.001*.

## Discussion

The aim of this work was to assess the effects of a 9 week computerized program on selective attention in a group of young soccer players. To this end, an intervention was carried out using the software Rejilla 1.0 during 27 sessions and 9 weeks on the experimental group. During the program, three evaluations were carried out using the computerized tool but with different exercises from those of the intervention. Likewise, the D2 test was used before and after the training program to evaluate in a complementary way the changes produced in this cognitive capacity. The results have highlighted differences between the two groups, suggesting positive effects on the experimental group and satisfying the research objectives.

First, the scores recorded from Rejilla 1.0 exercises have highlighted changes in the hit variable in the experimental group, although not in errors significantly. The control group did not show important changes, only some residual measures that do not show a clear trend and that could probably be caused by chance or by the learning effects of the instrument. This suggests, first, what previous research has pointed out, indicating that attention is a capacity that can be trained (Wass et al., 2011; Posner et al., 2015; Olfers and Band, 2018). Specifically, this work provides the possibility to evaluate this tool as an appropriate way for this purpose, adding to other instruments that had previously been used for the training and assessment of attention (Hernández-Mendo and Ramos-Pollán, 1995a,b; Chamberlain et al., 2011; Montani et al., 2014; Kirk et al., 2016).

However, the effects produced in the experimental group could be due to the learning of the computerized tool itself, which needs to be contrasted with other criteria. Although the evaluation and intervention have been performed on different exercises, the execution procedures are similar and could be influencing the results. Evaluations using the D2 test have made it possible to analyze this phenomenon. The results of this test have indicated significant changes in the experimental group. In addition, differences between groups have been accentuated in measures such as responses, hits, concentration, or attention. Therefore, the results suggest the effectiveness of the intervention program and reinforce the use of this type of intervention to improve this cognitive capacity (Reid et al., 2009; Rabiner et al., 2010; Bogdanova et al., 2016).

These findings have relevant implications in sports contexts, given previous studies that have shown the importance of attention to the performance of athletes, including soccer players (Weinberg and Gould, 2010; Carraça et al., 2018; Love et al., 2018; Roca et al., 2018). In fact, previous literature has shown that better overall cognitive functioning could contribute to improving the performance of athletes (Verburgh et al., 2014; Huijgen et al., 2015; Romeas et al., 2016; Fink et al., 2018). Therefore, such interventions could improve their skills to act during the course of the game. Improving cognitive functioning could help you react better to game situations that require effectively perceiving, issuing a quick response, discriminating against different stimuli, or deciding on a response. In sports like soccer, which are very variable, training this type of ability could allow for greater success. For this reason, using this type of training in a complementary way to other habitual routines in soccer could increase the preparation of the sportsmen to increase the efficiency in their game and, by extension, the performance of the team. In addition, the use of technology can increase the motivation of users to perform training tasks, being able to adapt for devices such as mobile phones or tablets easily accessible to young athletes who are accustomed to their use (Bordignon and Iglesias, 2016; De La Torre-Salazar et al., 2017). In addition, this type of tool facilitates the fast storage and analysis of the data obtained, allowing the technical team to easily monitor the progress of athletes.

This study has a number of limitations. On the one hand, the intervention time is only 9 weeks, which may not be preventing us from seeing more robust results on some measures. More protracted programs would probably offer more significant changes in the measures of attention assessed. On the other hand, it would be necessary to extrapolate this program to other populations, with different characteristics such as age, gender, category, or level of studies, in order to analyze whether these variables would modulate the results found. Finally, it would be interesting to use other complementary instruments such as criteria to contrast the evaluated measures and to observe more clearly the changes in the constructs under study.

In any case, the present study provides data that help to highlight the usefulness that computerized training in sportsmen and sportswomen could have for improving their cognitive functioning, and its possible implications in this type of context. In addition, it offers a versatile tool that can be used in sports sciences as a complement to other instruments that have already been used and that can be used to provide more resources to professionals working in this field.

## Data Availability Statement

The datasets generated for this study are available on request to the corresponding author.

## Ethics Statement

The studies involving human participants were reviewed and approved by Ethics Committee of the University of Malaga (19-2015-H). Written informed consent to participate in this study was provided by the participants' legal guardian/next of kin.

## Author Contributions

AH-M, VM-S, RR, JM-B, RJ-R, and FG-G participated in the study design and data collection, performed statistical analyses and contributed to the interpretation of the results, wrote the manuscript, and approved the final manuscript as presented. RR, AH-M, and FG-G conceived the study and participated in its design and coordination. AH-M, VM-S, RR, JM-B, RJ-R, and FG-G contributed to the interpretation of the results, reviewed, and provided feedback to the manuscript. All authors made substantial contributions to the final manuscript.

### Conflict of Interest

The authors declare that the research was conducted in the absence of any commercial or financial relationships that could be construed as a potential conflict of interest.

## Supplementary Material

Supplementary Video S1 This video presents an example of the programming of some of the exercises presented to young footballers. For more information about the program you can consult the article that presents the training program of the attention that appears in the article Hernández-Mendo et al. (2012). The Grid program can be downloaded from the online Psychosocial Assessment Platform MenPas (www.menpas.com ->Work Area I ->Attention->Download Grid). It is free and is distributed free of charge.
